# Harnessing Big Data in Critical Care: Exploring a new European Dataset

**DOI:** 10.1038/s41597-024-03164-9

**Published:** 2024-03-28

**Authors:** Niklas Rodemund, Bernhard Wernly, Christian Jung, Crispiana Cozowicz, Andreas Koköfer

**Affiliations:** 1 Department of Anesthesiology, Perioperative Medicine and Intensive Care Medicine, Paracelsus Medical University Salzburg, Salzburg, Austria; 2 Department of Internal Medicine, General Hospital Oberndorf, Teaching Hospital of the Paracelsus Medical University Salzburg, Oberndorf, Austria; 3 Center for Public Health and Healthcare Research, Paracelsus Medical University Salzburg, Salzburg, Austria; 4 Division of Cardiology, Pulmonary Diseases, Vascular Medicine Medical Faculty, University Dusseldorf, University Hospital Dusseldorf, Dusseldorf, Germany

**Keywords:** Clinical trial design, Experimental models of disease

## Abstract

Freely available datasets have become an invaluable tool to propel data-driven research, especially in the field of critical care medicine. However, the number of datasets available is limited. This leads to the repeated reuse of datasets, inherently increasing the risk of selection bias. Additionally, the need arose to validate insights derived from one dataset with another. In 2023, the Salzburg Intensive Care database (SICdb) was introduced. SICdb offers insights in currently 27,386 intensive care admissions from 21,583 patients. It contains cases of general and surgical intensive care from all disciplines. Amongst others SICdb contains information about: diagnosis, therapies (including data on preceding surgeries), scoring, laboratory values, respiratory and vital signals, and configuration data. Data for SICdb (1.0.6) was collected at one single tertiary care institution of the Department of Anesthesiology and Intensive Care Medicine at the Salzburger Landesklinik (SALK) and Paracelsus Medical University (PMU) between 2013 and 2021. This article aims to elucidate on the characteristics of the dataset, the technical implementation, and provides analysis of its strengths and limitations.

## Background & Summary

In the past decade, the momentum of epidemiological research in the medical scientific field has surged, primarily due to the utilization of large-scale datasets. This rapid progress has been facilitated by advancements in statistical methodology and computing power, enabling researchers to explore a wide range of critical clinical healthcare questions. These encompass various aspects such as practice patterns, temporal trends, healthcare disparities, cost of care, rare events, and patient harm^1^. More recently the interest in hospital based, routinely collected clinical patient data has significantly grown. In particular the critical care environment, offering information from patients under rigorous monitoring and stringent documentation has evolved to a valuable source of highly granular, real-world data^2,3^. As a result, several large critical care datasets are publicly available containing this longitudinal information. Most of these datasets build upon the original idea of the Medical Information Mart for Intensive Care (MIMIC) dataset, a waveform database with demographics digitally transcribed from paper records for over 90 patients^4^. Ultimately, MIMIC experienced various updates and enhancements as well as a significantly increase in sample size and breadth of information. Being meanwhile sourced from various digital information systems and from different institutions, MIMIC in its most recent version has been released as MIMIC-IV lately^5^. Subsequently, MIMIC has been complemented by another multicenter, US dataset (eICU-CRD), a Chinese Pediatric Intensive care dataset (PIC), and most recently by two European single center databases (HiRID, and Amsterdam UMCdb)^6^. Nevertheless, high resolution data, having more than one entry per hour, remains relatively scarce. HiRID contains admissions from 2005 to 2016 at Bern University Hospital, Switzerland, making it one of the most up to date high-resolution ICU datasets available^7^. Ultimately, high resolution is essential for the effective utilization of any artificial intelligence (AI) applications. As publications using AI in intensive care medicine, particularly machine learning (ML) algorithms, had an almost exponential growth over the last years, with a growth rate of 3.93% from 2011 to 2015, 52.1% from 2016 to 2020, and a remarkable 120.3% in 2022 alone, the need for additional high resolution data in critical care is immanent^8^.

To address this need and to overcome one of the main drawbacks of almost all current AI-based research in critical care, namely relying repeatedly on the same dataset, we have created the Salzburg Intensive Care database (SICdb)^9,10^. SICdb is a publicly available, high resolution critical care dataset that enhances the availability of medical data to the public. SICdb is fully funded by the Department of Anesthesiology, Perioperative Medicine and Intensive Care Medicine, Paracelsus Medical University Salzburg, Austria. Full funding is currently guaranteed until 2028. In this article, we offer a thorough exploration of the data structure, patient cohort, and additional background information embodied within SICdb (1.0.6). SICdb covers almost a decade of admissions at four different intensive care units (ICUs) at one single tertiary care institution of the Department of Anesthesiology and Intensive Care Medicine at the Salzburger Landesklinik (SALK) and Paracelsus Medical University (PMU) between 2013 and 2021. With a total of 27,386 admissions SICdb is amongst the largest datasets available worldwide. With up to date, high resolution data, the inclusion of data from preceding surgeries/procedures, and annual updates, SICdb has the potential to advance data science-based research.

## Methods

### Data Acquisition

#### Cohort

SICdb includes patients admitted to one of the four participating intensive care and stepdown units at the University Hospital Salzburg, from 2013 to 2021. University Hospital Salzburg is an Austrian tertiary care center responsible for population of approx. 650,000 in the greater area of Salzburg and the neighboring countries providing a total of 58 ICU and IMC beds of which 41 were used to generate data for SICdb. SICdb primarily contains cases of surgical and general intensive care medicine from all surgical disciplines. The most common procedures include cardiac surgery, followed by vascular surgery, general surgery, and trauma/orthopedic surgery. SICdb was approved by the State Ethic Commission of Salzburg, Austria. (EK Nr: 1115/2021). Due to the anonymous nature of the data and the clinic’s data usage agreement, explicitly allowing patient data to be utilized for scientific purposes, the ethics committee waived the need for individual consent. SICdb is subject to the regulations of the European General Data Protection Regulation (GDPR) in its current form (https://eur-lex.europa.eu/eli/reg/2016/679/oj). Data access to SICdb is governed by a Data User Agreement (DUA). Due to the low number of underaged patients and to minimize therefore any risk of reidentification, SICdb excludes all patients under the age of 18.

### Data sources

SICdb contains data from various sources. Most notably from MetaVision® (iMDSoft, Tel Aviv, Israel), ORBIS® (Dedalus Healthcare GmbH, Bonn, Germany), and Statistics Austria (Austrian Federal Statistical Office (German: Bundesanstalt Statistik Österreich) the country’s agency for collecting and publishing official statistics related to Austria). The primary data source was the MetaVision® ICU patient data management system (PDMS). MetaVision® consolidates a variety of data, encompassing monitor signals, laboratory parameters, medication details, fluid balances, and respirator settings, among others. The export from ORBIS® (Dedalus HealthCare) electronic health record contained mainly ICD10 diagnosis codes, duration of hospital and in-hospital mortality data. All ICD10 codes have been encoded within the first two days after admission. Data on long term mortality was provided by Statistics Austria, matching patients by clear name and birth date.

### Data processing

The primary source of SICdb has been MetaVision®. The MetaVision® database was provided as a MSSQL (Microsoft SQL Server) database in a safe virtual environment on the premises of Salzburg University Hospital. The data was extracted, restructured and deidentified. To maximize anonymity, a cryptographically secure random number generator was used to reassign all identifiers. Due to data size and structure the export process was expected to take a significant amount of time. Therefore, it was designed to be interruptible and transactionally safe. The process complied to the ACID (atomicity, consistency, isolation, durability) principle of database transactions. The export process allows for incremental updates, enabling the incorporation of new data on an annual basis. This intermediate stage raw data then was restructured into a scientifically usable dataset. The data was exported to RFC 4180 comma-separated value files and compressed using gzip^11,12^. The file size containing the raw minute data exceeded the limits allowed on the PhysioNet repository. Thus, it was preserved as a continuous sequence of IEEE 754 encoded floating-point numbers, capable of holding up to 60 values per row. This significantly decreases dataset size. The dataflow during the export process has been described previously^10^.

**Fig. 1:**
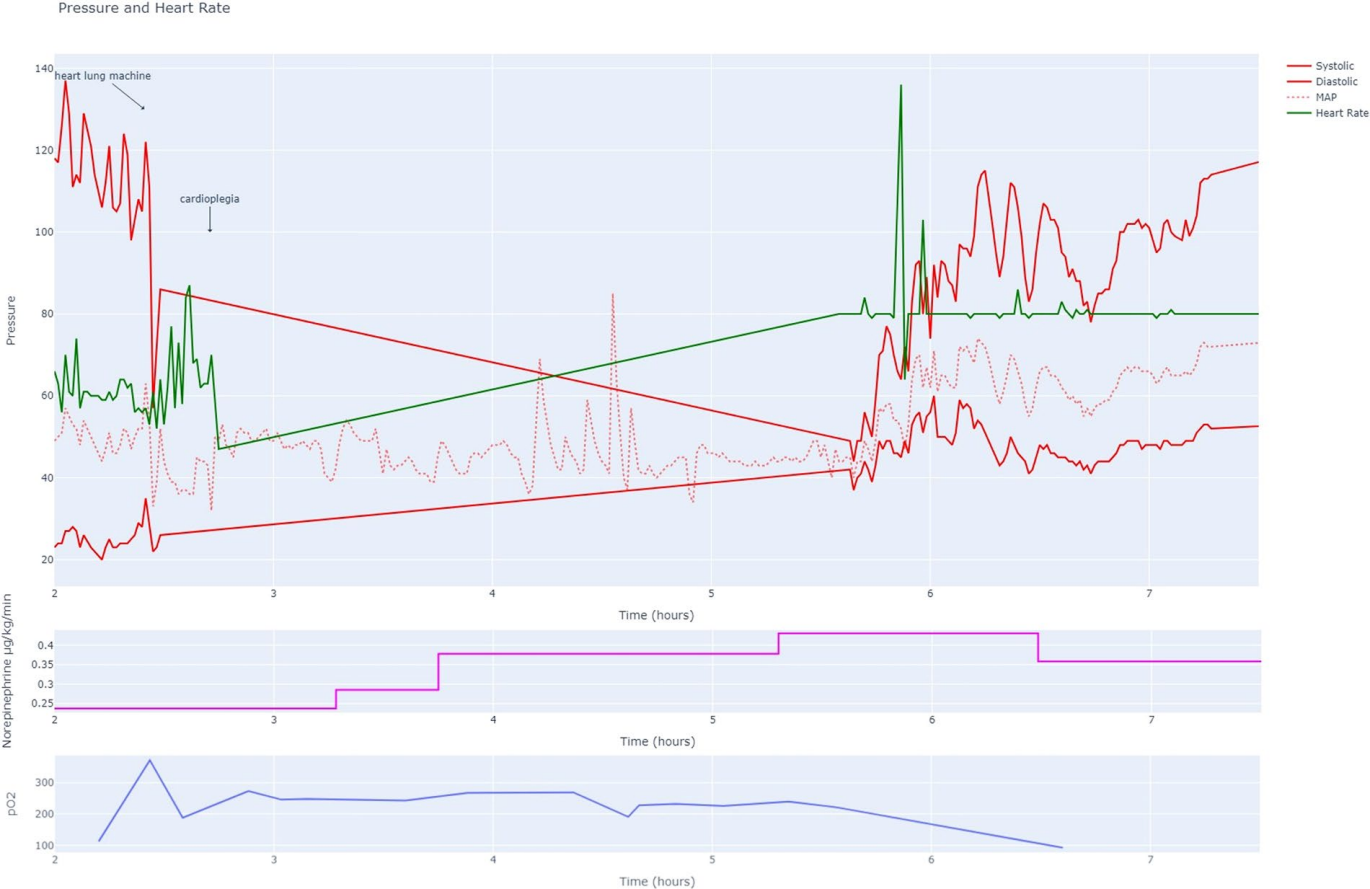
A comprehensive depiction of a typical heart surgery patient using cardiopulmonary bypass (CPB) obtained from SICdb. The dataset offers high level of granularity, enabling detailed analysis of vital signs, medication administration, and laboratory findings throughout the surgical procedure. The provided sample demonstrates two critical events: the time of cross clamping, characterized by distinct changes in pulse signals with the absence of systolic or diastolic pressure, and the application of cardioplegia, resulting in the loss of the ECG heart rate signal. In the middle panel of the figure, the continuous administration rate of noradrenaline is presented in micrograms per kilogram per minute (mcg/kg/min). The figure offers insights into the dosage and regulation of noradrenaline during an exemplary cardiac surgical procedure. In the lower panel, the oxygen measurements obtained from arterial blood gas analysis are provided, giving essential information regarding oxygen levels. (BPM: Beats Per Minute, HR: Heart Rate, MAP: Mean Arterial Pressure).

Tables 1 and 2 intend to give an exemplary insight in the data provided in the dataset. We expressed continuous data points as median ± interquartile range or as mean ± standard deviation, as appropriate. Categorical data were stated in numbers (percentage). Statistical analyses were performed using MySQL (version 8.0.29), R (version 4.1) and Python’s SciPy library, respectively. Visual representations and plots (Fig. 1) were generated using the Plotly library in Python.

**Table 1:** Main demographic data of the patients included in SICdb. Main diagnosis and basic mortality rates are displayed.

Admissions	27,386
Age, mean (SD)	65.91 (15.71)
Female gender as documented n (%)	10,288 (37.5)
Austrian address n (%)	25,466 (92.9)
Days of icu stay, mean (SD)	3.50 (6.50)
In-hospital mortality^a^ n (%)	2,186 (7.9)
Out-of-hospital mortality^b^ (%)	5,093 (18.5)
SAPS3 mean (SD)	44.6 (14.8)
Mechanically ventilated >24 h n (%)	2,406 (8.79)
Renal replacement therapy (RRT) n (%)	1,027 (31.75)
*Most common ICD 10 diagnosis*	
I25.X (ischemic heart disease) n (%)	1,930 (7.05)
I70.X (atherosclerosis) n (%)	1,352 (4.94)
I35.X (non-rheumatic aortic valve disease) n (%)	1,186 (4.33)
I71.X (aneurysm or dissection of aorta) n (%)	863 (3.15)
I65.X (occlusion of cerebral artery n (%)	741 (2.71)

^a^Includes the Mortality during the stay at the Hospital in which the admission to ICU occurred.

^b^Includes data on long term mortality reported by Federal Austrian Statistics Office as of 01/01/2022. The observation period for mortality is one year from the time of admission.

**Table 2:** A compilation of common laboratory parameters, accompanied by their respective median values and interquartile ranges.

n	Parameter	Median	IQR	Unit
172,782	Erythrocytes	3.1	[2.7, 3.7]	10^6/µL
172,745	Leucocytes	9.79	[7.36, 13.15]	10^3/µL
172,723	Hemoglobine	9.2	[8.1, 10.9]	g/dL
172,636	Thrombocytes	218	[153, 310]	10^3/µL
168,786	Urea	39	[27, 62]	mg/dL
167,245	Creatinine	0.9	[0.7, 1.3]	mg/dL
164,850	C-reactive Protein	7.2	[2.5, 14.3]	mg/dL
160,998	Calcium	2.11	[2.01, 2.21]	mmol/L
156,193	Sodium	139	[137, 142]	mmol/L
151,483	Activated Partial Thromboplastin Time	36	[31, 44]	seconds
140,257	Prothrombin Ratio	82	[69, 95]	%

### De-Identification

The anonymization methods followed the regulations of the GDPR. The deidentification strategy also complies with the US regulations for health data, the Health Insurance Portability and Accountability Act (HIPAA) Safe Harbor^13^. HIPAA specifies 18 variables, including but not limited to names, addresses, dates, social security, and medical record numbers, all of which have been excluded from the dataset. No personal data other than those being crucial for export, processing or the consecutive research were accessed. All free-text fields have been removed to reduce the risk of exposing any personal health information (PHI). Elements that may be important for research (e.g.: age, sex and weight) have been grouped into bins of 5, with ages over 90 placed in a final bin to increase k-means anonymity^14^. As defined in the GDPR all potential identifiers were reassigned with cryptographically safe random numbers in first data processing step. As any date and time information has to be considered to be PHI^15^, all dates and times during the stay were recalculated to a relative time in seconds starting from admission^15^. The absolute admission time is stored within the lookup database for further processing and is not published. The year of admission was added to the dataset as it may be important for retrospective analysis and is not considered PHI.

## Data Records

SICdb is accessible on PhysioNet for credentialed users only^9^. The dataset, version 1.0.6, includes data on 27,386 admissions at the Department of Anesthesiology and Intensive Care Medicine. However, it should be noted that there is an additional ICU at Salzburg University Hospital (SALK) specifically catering to patients following conventional non-surgical cardiac procedures and those with internal medical conditions. Unfortunately, due to technical constraints arising from a non-compatible health record system, data from this particular unit could not be included in the SICdb. As a result, SICdb may exhibit a relative overrepresentation of patients who underwent surgery or experienced trauma. The base table, ‘cases’, contains one entry per admission. ‘cases’. ‘CaseID’ is the primary identifier which is used in all related tables. Each ‘CaseID’ corresponds to an admission within the MetaVision® PDMS, encompassing pre-surgery, surgery, and intensive care data. For alignment with intensive care datasets that exclude surgery data, the file ‘cases’. ‘ICUOffset’ indicates the time, in seconds, from the PDMS admission to the initial admission to intensive care unit. Basic demographic analysis and ICD-10 diagnose codes are presented in Table 1.

In total SICdb, version 1.0.6, contains over 1.5 billion signal data entries from several sources like monitors and respirators, disclosed once-per-minute. The laboratory data includes the central laboratory data, and data of most point-of-care analytical devices in use. It is saved in the ‘laboratory’ table. Applied medication and fluids are included in the ‘medication’ table. Signal data is distributed in the ‘data_float_h’ table.

### Timing information

Date information, other than admission year, has been removed from the dataset. Each data item has an ‘offset’ field, that contains time in seconds from admission to PDMS. This may include preceding pre-surgery optimization and surgery. If a patient has two distinct hospital stays, a new CaseID is generated, with the offset again relative to the PDMS for this specific admission. The field ‘OffsetAfterFirstAdmission’ specifies the time elapsed, in seconds, from the initial hospital admission. Figure 1 shows timing, selected medication and hemodynamic parameters of one example case during cardiac surgery using cardiopulmonary bypass (CBP).

### Encoding

All categorical data is encoded. The reference is found in ‘d_references’. ‘ReferenceGlobalID’ relates to every encoded field of the dataset, serving as a dictionary. Additionally, if applicable, the unit of measurement is provided within this table.

### Medication data

The medication table contains information on applied medication and fluids. Field ‘Offset’ reflects, similar to other tables, the time in seconds from admission. The variable ‘OffsetDrugEnd’ denotes the end time of a medication application in seconds. For bolus applications, the time of application is defined as 60 seconds, and the IsSingleDose field is set to 1. ‘Amount’ is the total given dosage, ‘AmountPerMinute’ is a convenience field simplifying queries of continuous dosages. If applicable, the unit of measurement can be found in ‘d_references’ where ‘medication’. ‘DrugID’ corresponds to ‘d_references’ ‘ReferenceGlobalID’

### Laboratory data

The laboratory table encompasses 17,702,557 laboratory readings from 426 distinct laboratory parameters, sourced both from point-of-care devices and the central clinic laboratory. Analogous to the ‘medication’ table, the ‘Offset’ column represents the time elapsed since admission, measured in seconds. The names and units of measurement, are to be found in the ‘d_references’ table. ‘Laboratory’. ‘LaboratoryID’ aligns with the ‘d_references’.‘ReferenceGlobalID’. Due to the high daily sampling rate, 75.8% (n = 13,430,538) of measurements are derived from blood gas analysis (BGA). Among the 382 non BGA laboratory parameters in the dataset, those with the highest number of entries are blood count (hemoglobin and hematrocrit), electrolytes, creatinin, bun, INR, aminotransferases and bilirubin, respectively. Table 2 details the most significant of these frequently logged parameters, presenting their count, median and interquartile ranges. Table 2 lists a selection of important recorded parameters.

### Data tables

For convenience, laboratory and medication data is distributed within separate tables. However, huge amounts of entries are shipped in generic data tables, defined by the data type. The larges data table is ‘data_float_h’, which contains hourly aggregated data (mean) and provides raw data. Due to the 10-gigabyte file size constraint of the PhysioNet repository during its primary release, it wasn’t feasible to format the raw data as one row per item. Instead, the detailed data is serialized as a continuous sequence of IEEE 754 encoded floating-point numbers, capable of holding up to 60 values per row and provided in the ‘data_float_h’.‘rawdata’ field. This substantially reduces the file size. When applied in a relational database setting, both database and index sizes are minimized, leading to decreased overhead and enhanced query speeds. The largest part of ‘data_float_h’ is monitor signal data, which has been collected at a frequency of once per minute. The dataset comprises approximately 270 patient years of accumulated data, wherein the most prevalent 8 vital signals, encompass a total of 884,714,655 data points. On average, this translates to 48.06 entries per hour of observation time (Table 3). Deserialization scripts are provided in our online documentation (https://www.sicdb.com/Documentation/Main_Page) and online code repository (https://github.com/nrodemund/sicdb), respectively.

**Table 3:** The most common vital parameters recorded in SICdb.

Name	Count^a^	Samples Per Hour^b^
Heart Rate (ECG)	125,240,471	54.43
SpO2	120,351,010	52.31
Heart Rate (SpO2)	113,338,064	49.26
Mean arterial pressure (MAP)	107,141,596	46.57
Arterial pressure (systolic)	106,287,594	46.19
Arterial pressure (diastolic)	106,251,685	46.18
Temperature	106,953,492	46.48
Respiratory Rate	99,150,743	43.09

^a^Refers to the absolute number of recorded data points.

^b^Represents the rate of entries made over the entire observation period, measured in data points per hour.

### Scores and diagnostic codes

SICdb includes several scores from different sources. In SICdb The Simplified Acute Physiology Score III (SAPS III) is used for general survival prediction^16,17^. Additionally, data from heart surgery patients are more detailed and includes the EURO Score II for a priori mortality prediction in cardiac surgery^18^. A valid ICD10 code representing the primary diagnosis was a prerequisite for inclusion in SICdb. 0Hence, ICD10 is available for every patient entry.

## Technical Validation

A row-based versioning and validation system has been established to prevent incomplete data in case of interruption during the long running export and processing procedure. Software and scripts used for dataset processing follow recommended best practices in scientific computing^19^. Unit tests ensured consistency of row-based versioning before each release. The resulting database was cross-validated with secondary export from ORBIS®, ensuring completeness.

However, SICdb represents a “real world” dataset, making it susceptible to potential human errors. While the majority of the data was collected electronically, certain entries, such as patient data (e.g., weight and height), form data (e.g. premedication and preexisting diseases and conditions), and applied medication were also recorded manually. We deliberately choose not to preprocess the majority of this data. This allows ML and deep learning models to handle and learn from real-world data to be effective and robust in any practical applications. Additionally, we found that most models have their own preprocessing and may not be compatible with our individual methods. However, there are three exceptions, namely: height, weight and biological sex. Those were identified as utterly important for retrospective analysis. Unknown biological sex only occurred in 7 cases and was manually corrected in version 1.0.4. Height and weight, which is entered manually in MetaVision®, failed the plausibility analysis in 207 cases. Most common errors were flipped height/weight values and missing zeroes. Implausible values were corrected by manual lookup in medical archive. In version 1.0.6 the original values were added to enable checking model performance using uncorrected data.

Lastly, we’ve added a low amount of processed additional information, most notably fields KDIGO_AKI_48 and KDIGO_AKI_168. The algorithms for these fields are documented, versioned and disclosed on our code repository.

### Raw data validation

The raw data used to create SICdb contained a significant number of invalid items. This included, among others, accidentally created entries, test data, PDMS development data and duplicates. Therefore, we developed a strategy to identify low quality entries: First, we cross matched the dataset with ICD10 scores as this information was sourced from a separate database (ORBIS®) to ensure that the patient identifier was accurate and to eliminate any invalid or testing cases. Second, all cases with any implausible data were removed. This included entries without signal data and/or a missing (mandatory) admission forms. We found that those entries most commonly represented duplicates.

### Mortality validation

**Fig. 2:**
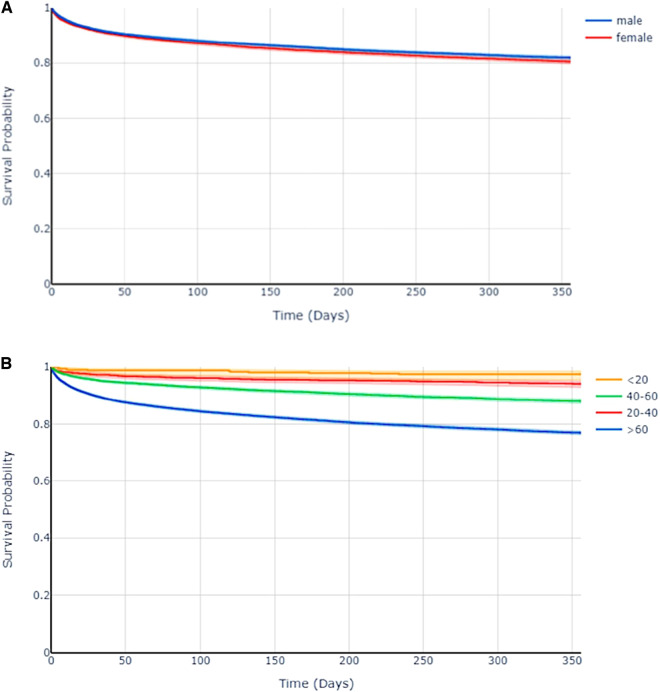
(**A** and **B**) The graph shows a survival analysis separated by different age groups (**A**). Indicating the expected worse long-term survival of old and very old patients. Additionally, the survival analysis separated by sex is displayed in (**B**). (The shaded area in both graphs represents the 95% confidence intervals).

To identify mortality two strategies were applied. First, we identified in-hospital death by merging the PDMS (MetaVision®) and ORBIS® records. Second, we added mortality data provided by Statistic Austria. However, as due to the fact that University Hospital Salzburg is a superregional center taking care for non-Austrian patients too, as well as due to the geographic location close to Germany we needed to identify the percentage of patients not reported to the Federal Austrian Statistics Office. We have identified 25,466 patients with an Austrian home address versus 1,200 with a non-Austrian address. Among the Austrian patients, the one-year mortality rate is 19.4% (n = 4,939), while in the non-Austrian group it is 8.0% (n = 154). The mean time to death, for those patients dying within the first year after admission, is 89.94 days (±98.92 days) for Austrians and 44.60 days (±73.84 days) for non-Austrians, respectively. Figure 2A and B show the long-term survival after admission to the ICU stratified by age groups and sex. However, this analysis is somewhat biased by a higher loss-of-follow-up rates for non-Austrian patients.

## Usage Notes

To access SICdb (1.0.6), contributors’ approval must be obtained and a specific research question must be provided. Additionally, it is required to be a credentialed PhysioNet user, which requires an identity check, the prove of an appropriate medical data usage training course and signing a data use agreement. The data may only be used for the sole purpose of lawful use in scientific research. Sharing the data with third parties is prohibited. We would like to remind all potential users that the dataset contains sensitive clinical data. As such, all data must be treated with the utmost care and respect. Any attempt to identify individual patients using this dataset is illegal by European law.

Documentation on the dataset is available online and via the PhysioNet repository^10^. Documentation contains a table schema and detailed descriptions of all fields and the data. However, the most up-to-date information can be found on the SICdb website (https://www.sicdb.com/Documentation/Main_Page) Additionally, we have created a repository on GitHub (https://github.com/nrodemund/sicdb) to share code and facilitate discussions about the dataset. GitHub can also be utilized for bug reports, suggestions, and contributions.

## Data Availability

All publicly available code can be accessed from the SICdb GitHub Code Repository (https://github.com/nrodemund/sicdb). However, due to the partial use of the code to appropriately remove sensitive patient information in accordance with HIPAA regulations, not all codes are fully publicly accessible. Furthermore, the GDPR restricts the sharing of certain code components to ensure the highest level of anonymization.
